# Assessment of metabolic syndrome predictors in relation to inflammation and visceral fat tissue in older adults

**DOI:** 10.1038/s41598-022-27269-6

**Published:** 2023-01-03

**Authors:** Anna Tylutka, Barbara Morawin, Łukasz Walas, Marta Michałek, Anna Gwara, Agnieszka Zembron-Lacny

**Affiliations:** 1 Department of Applied and Clinical Physiology, Collegium Medicum University of Zielona Gora, 28 Zyty Str., 65-417 Zielona Gora, Poland; 2 Institute of Dendrology, Polish Academy of Sciences, Parkowa 5, 62-035 Kórnik, Poland; 3 Student Research Group, Collegium Medicum, University of Zielona Gora, 28 Zyty Str., 65-417 Zielona Gora, Poland; 4 Department of Nursing, Collegium Medicum, University of Zielona Gora, 28 Zyty Str., 65-417 Zielona Gora, Poland

**Keywords:** Cytokines, Inflammation

## Abstract

The diagnosis of metabolic syndrome (MetS) focuses on the assessment of risk factors such as insulin resistance, dyslipidemia, central adiposity and elevated blood pressure. Evidence suggests that markers of systemic inflammation may also be included in the definition of MetS and play some role in its pathogenesis. The study was designed to evaluate low-grade inflammation status in older adults with MetS in relation to increased body fat tissue and an attempt was made to evaluate new predictors for MetS through the analysis of the ROC Curve. Ninety-six middle-aged (69.2 ± 4.9) individuals from University of Third Age (women *n* = 75 and men *n* = 21) were allocated to two groups: without metabolic syndrome (*n* = 37) and with metabolic syndrome (*n* = 59) according to International Diabetes Federation criteria in agreement with American Heart Association/National Heart, Lung and Blood Institute 2009. Participants’ current health status was assessed using medical records from a routine follow-up visit to a primary care physician. Statistical analysis was performed using R studio software. Depending on the normal distribution, ANOVA or the Kruskal–Wallis test was used. The optimal threshold value for clinical stratification (cut-off value) was obtained by calculating the Youden index. The AUC was observed to be the highest for a new anthropometric index i.e. lipid accumulation product (0.820). Low-grade inflammation dominated in MetS group (BMI 28.0 ± 4.4 kg/m^2^, WHR 0.9 ± 0.1, FM 24.7 ± 7.9 kg) where significantly higher values of TNF-α (*p* = 0.027) and HGMB-1 protein (*p* = 0.011) were recorded.The optimal threshold values for immunological indices assessed as new predictors of the metabolic syndrome were: 93.4 for TNF-α, 88.2 for HGMB-1 protein and 1992.75 for ghrelin. High AUC values for these indices additionally confirmed their high diagnostic usefulness in MetS.

## Introduction

The incidence of overweight and obesity has increased dramatically over the past few decades. Obesity is strongly correlated with a significant number of metabolic disorders, including insulin resistance, atherogenic dyslipidemia, non-alcoholic fatty liver disease or metabolic syndrome (MetS)^1^. MetS is a known risk factor for type 2 diabetes, cardiovascular mortality^2^ or persistent low-grade chronic inflammation. There are several criteria for metabolic syndrome diagnosis and the International Diabetes Federation criteria are focused on the central obesity. So far, body composition variables used as predictors ofmetabolic syndrome have included the body mass index (BMI), waist circumference (WC), or waist to hip ratio (WHR)^3^. Kahn proposed the use of lipid accumulation product (LAP), a novel index of central lipid accumulation, to predict the risk of MetS^4^. Adipose tissue is not able to buffer dietary lipids and it leads to ectopic deposition of fat in other organs like the liver, heart or skeletal muscles. Lipotoxicity in adipose tissue increases the volume of reactive oxygen species (ROS) the excess of which causes cellular damage and leads to chronic inflammation^5^. In both conditions, obesity and MetS, the adipose tissue is altered. Stress and inefficient oxygenation cause adipocytes to undergo apoptosis. This initiates the accumulation and activation of macrophages with an M1 (proinflammatory) phenotype as well as a decrease of the M2 (anti-inflammatory) phenotype^6^. Studies have shown a relationship between the concentration of tumor necrosis factor (TNF-α) and the intensity of inflammatory processes in older adults with increased adipose tissue^7,8^. TNF-α concentration is correlated with impaired insulin receptors and it causes a decrease in GLUT-4 translocation to the plasma membrane, which leads to insulin resistance^9^. TNF-α overexpression plays a crucial role in the development of insulin resistance, which is an important component of MetS, and the available systematic review suggested that TNF-α could be an early biomarker for MetS detection^10^. A similar effect on the insulin receptor is produced by interleukin-6 (IL-6) the cytokine which inhibits the expression of insulin receptors and reduces adiponectin level^9^. IL-6 is strongly correlated with obesity and is called a marker of visceral tissue because it is the one that releases considerably more cytokines than subcutaneous adipose tissue^11^. Proinflammatory cytokines like TNF-α, IL-6 orinterleukin-1β (IL-1β) and metabolites from damaged cells induce HMGB-1 translocation from the nucleus to cytosol. This protein is identified as an important stimulus of inflammation at the site of the injury. Consequently, it is understandable that HMGB-1 level is higher in obesity and metabolic syndrome, as in these diseases inflammation initiated by lipotoxicity and ROS cause cellular damage and macrophages activation^12^. The anti-inflammatory interleukin-13 (IL-13), in turn, appears to be the main regulator of glucose metabolism as it inhibits the transcription of hepatic genes encoding key gluconeogenic enzymes, such as PEPCK and G6P. However, the molecular mechanisms underlying the regulation of IL-13 expression and development of the metabolic syndrome are, as yet unclear^13^.

Chronic low-grade inflammation and immune system dysfunction observed in both obesity and MetS lead to worse outcomes, enhance the negative impact of obesity and cause a higher cardiovascular risk. Therefore, the aim of our study was to evaluate a low-grade inflammation status in older adults with the metabolic syndrome in relation to an increased body fat tissue. Moreover, an attempt was made to evaluate new predictors for the metabolic syndrome assessment.

## Materials and methods

### Participants

The study participants (123 individuals) were recruited from the University of the Third Age (U3A) in Zielona Gora. This organization promotes active lifestyles among older adults over 60 years of age by involving them in many educational programmes. The participants’ current health status was assessed using the medical records from routine follow-up visits to a primary care physician. The inclusion criteria included 60 years of age or older, the same access to medical healthcare provided by the same medical center, and no hospitalization in the last 6 months prior to the study. The exclusion criteria included autoimmune diseases and acute infectious, neurodegenerative and oncological diseases, uncontrolled hypertension, musculoskeletal disorders and an implanted pacemaker. Twenty-seven older adults were excluded from further participation due to hospitalization (*n* = 5), common cold (*n* = 8) or demotivation or unavailability of recruited individuals to participate in the study (*n* = 14). Taking into account the total number of people attending U3A (*n* = 950), the confidence level 95% and the margin of error of 10%, the optimal sample size should be at least *n* = 88 individuals. Having applied the inclusion and exclusion criteria, eventually 96 subjects (women *n* = 75 and men *n* = 21) were enrolled in our study.

### Definition of metabolic syndrome and classification to the group

Metabolic syndrome was evaluated according to modified International Diabetes Federation (IDF) criteria in agreement with American Heart Association/National Heart, Lung and Blood Institute (AHA/NHLBI) of 2009^14^ including three of the following five abnormalities: (1) high blood pressure (SBP ≥ 130 mmHg, DBP ≥ 85 mmHg) or a diagnosis/treatment of hypertension; (2) hyperglycemia (fasting whole-blood glucose concentration ≥ 100 mg/dL) or pharmacotherapy of diagnosed diabetes; (3) low concentration of HDL-cholesterol < 40 mg/dL (1.0 mmol/L) for men and < 50 mg/dL (1.3 mmol/L) for women; (4) hypertriglyceridemia (TG ≥ 150 mg/dL); (5) abdominal obesity (WC > 94 cm for men and > 80 cm for women). Based on the criteria described above, 96 participants were divided into two groups: without MetS (*n* = 37) and with MetS (*n* = 59).

### Anthropometric measurements

Body composition analysis was performed using a non-invasive method by means of Tanita Body Composition Analyzer MC-980 (Tokyo, Japan) calibrated according to the manufacturer’s guidelines prior to each test session. The analyzer was medically certified as MDD CLASS IIa and NAVI: CLASS III, which are required if the equipment is to be used in medical applications. The patient’s body weight (BM), fat mass (FM) and fat-free mass (FFM) were assessed. Before the examination, the patients were informed on how to prepare themselves. The tests were performed on an empty bladder in the morning before blood sampling. The patients were informed that no alcohol or caffeine drinks should be consumed 24 h before the examination, that they should not engage in any physical activity directly before the examination, and preferably refrain from exercising for 12 h before the analysis. Duplicate measurements were made in the study participants standing upright and the average value was included for the final analysis according to our previous research by Tylutka et al.^15^. The values of BMI of 18.5 kg/m^2^ were defined as low; BMI between 18.5 and 24.9 kg/m^2^ was considered normal, the values between 25 and 29.9 kg/m^2^ were classified as overweight and BMI ≥ 30 kg/m^2^ was regarded as obese according to World Health Organization (WHO)^16^. Waist circumference [cm] was measured using a non-elastic tape at the midpoint between the last rib and the upper edge of the iliac crest at the end of the expiratory movement. The hip circumference (HC) [cm] was also measured using a non-elastic tape around the midline of the greater trochanter. WHR was defined as WC [cm] /HC [cm]. Visceral adiposity index (VAI) was calculated according to Qi et al.^17^

$${\text{VAI }} = \, \left[ {{\text{WC}}/\left( {{39}.{68 } + { 1}.{88 } \times {\text{ BMI}}} \right)} \right] \, \times \, \left( {{\text{TG}}/{1}.0{3}} \right) \, \times \, \left( {{1}.{31}/{\text{HDL}} - {\text{C}}} \right)\left( {{\text{Male}}} \right),$$

$${\text{VAI }} = \, \left[ {{\text{WC}}/\left( {{36}.{58 } + { 1}.{89 } \times {\text{ BMI}}} \right)} \right] \, \times \, \left( {{\text{TG}}/0.{81}} \right) \, \times \, \left( {{1}.{52}/{\text{HDL}} - {\text{C}}} \right)\left( {{\text{Female}}} \right).$$

Lipid storage index (LAP) was calculated according to Kahn et al.^4^

$${\text{LAP}} = \left[ {{\text{WC}} - {65}} \right] \times {\text{TG}}\left( {{\text{Male}}} \right),$$

$${\text{LAP}} = \left[ {{\text{WC}} - {58}} \right] \times {\text{TG}}\left( {{\text{Female}}} \right).$$

Following a resting period of at least 15 min, blood pressure (BP) was measured on the right arm by automatic manometry using a GE Healthere (Germany) with an appropriate cuff size.

### Blood collection

Fasting blood samples were collected from the median cubital vein in the morning between 8.00 and 10.00 using S-Monovette tubes (Sarstedt AG & Co. KG, Nümbrecht, Germany). The whole blood samples were placed into specimen tubes containing EDTA and were immediately analyzed. The blood samples were centrifuged at 3000 rpm for 10 min, and aliquots of serum were stored at − 80 °C for the other biochemical analyses. The average intra-assay coefficients of variation (intra-assay CV) for the used ELISA kits were < 8%. All samples were analyzed in duplicate or triplicate in a single assay to avoid inter-assay variability. The procedures were conducted according to our previous research by Tylutka et al.^15^.

### Hematological variables

BM HEM3 3 diff analyzer from Biomaxima (Lublin, Poland) was used to assess the basic hematological parameters. The analysis included the assessment of the red blood cells counts (RBC), hemoglobin (HB), hematocrit (HCT), mean corpuscular volume (MCV), mean corpuscular hemoglobin (MCH), mean corpuscular hemoglobin concentration (MCHC), and white blood cells: white blood cell count (WBC), granulocytes (% GRA), lymphocytes (% LYM), and platelet count (PLT). The method of analyses of hematological parameters was based on previously reported methods by Tylutka et al.^15^.

### Biochemical variables

Serum triglycerides (TG), total cholesterol (TC), high-density lipoproteins (HDL), low-density lipoproteins (LDL) were determined using BM200 Biomaxima (Lublin, Poland). The non-HDL cholesterol was calculated by subtracting HDL from total cholesterol concentration. Oxidised low-density lipoprotein (oxLDL) was determined using ELISA kits from SunRed Biotechnology Company (Shanghai, China) with a detection limit at 30.3 ng/L. The analyses were conducted according to our previous research by Tylutka et al.^18^. The serum glucose level was evaluated by using commercially available reagents and mobile spectrophotometer DP 310 Vario II (Berlin, Germany). The insulin level was measured using a high sensitivity assay in duplicate by means of commercial kit from DRG International (Springfield Township, Cincinnati, OH, USA) with a detection limit of 0.001 μU/mL. HOMA index (Homeostatic Model Assessment Insulin Resistance) was calculated according to Sitar-Tǎut et al.^19^, HOMA-IR = insulin (μU/mL) × glycaemia (mg/dL)/405.

### Inflammatory variables

Tumor necrosis factor, interleukin-6, interleukin-8 and anti-inflammatory interleukin-13 were determined by using ELISA kits from SunRed Biotechnology Company (Shanghai, China). Adiponectin, leptin and ghrelin were determined by using ELISA kit from R&D system (USA), DRG International (USA) and SunRed Biotechnology Company (Shanghai, China). The adiponectin/leptin (Adpn/Lep) ratio was calculated according to Frühbeck et al.^20^. A proinflammatory protein high mobility group B1 (HGMB-1) was determined by using ELISA kits from SunRed Biotechnology Company (Shanghai, China) with detection limits of 0.526 ng/mL.

### Statistical analysis

All statistical analyses were performed using R system 4.2.1^21^. The data were described as measures of central tendency (mean and medians) and measures of dispersion [standard deviation (SD) or interquartile range (IQR)] for numerical variables. To check data normality, the Shapiro–Wilk test was applied. When the normal distribution was assumed, one-way ANOVA was used otherwise, the Kruskal–Wallis test was applied. Spearman rank correlation coefficient (r_s_) or Pearson correlation coefficient (R) was used to assess the agreement between metabolic syndrome and the continuous independent variables. The optimal threshold value for clinical stratification (cut-off value) was obtained by calculating the Youden index. Statistical significance was set at *p* < 0.05.

### Ethics declarations

All participants were informed of the aim of the study and gave their written consent for participation in the project. The protocol of the study was approved by The Bioethics Commission at Regional Medical Chamber Zielona Gora, Poland (N^o^21/103/2018) in accordance with the Helsinki Declaration.

## Results

### Body composition and basic group characteristics

The mean age of all the participants was 69.2 ± 4.9 with females being in the majority of the group (78%). The body mass index ranged from 18 kg/m^2^ to 44.8 kg/m^2^. In the group with MetS, 25% of the individuals were classified as overweight and approximately 51% were obese. In turn, in the patients without MetS as many as 68% of them had normal body weight and only 8% of the participants were classified as obese (Table 1). There were statistically significant differences between the groups in standard anthropometric indices that can be used in the diagnosis of the metabolic syndrome like FFM (*p* < 0.0001), FM (*p* < 0.001) or WHR (*p* < 0.0001). High systolic blood pressure, ≥ 130 mmHg, was recorded in ~ 40% of the patients without MetS and in ~ 70% of the MetS group.In turn, elevated diastolic blood pressure ≥ 85 mmHg prevailed in the group of patients with a diagnosed metabolic syndrome (44%).

**Table 1:** Body composition and basic group characteristics.

Variables	Without MetS *n* = 37		With MetS *n* = 59		*p*-value
	Mean ± SD	Med (iqr 25%-75%)	Mean ± SD	Med (iqr 25%-75%)	
Age [years]	67.9 ± 4.5	68.0 (65.0–70.0)	70.9 ± 4.9	70.0 (67.0–71.0)	0.030
Weight [kg]	62.8 ± 9.5	60.9 (56.0–66.5)	75.9 ± 13.8	73.1 (66.5–84.7)	< 0.0001
Height [cm]	160 ± 6.6	160 (154.5–162.5)	164.6 ± 8.1	163.0 (157.8–171.0)	0.007
BMI [kg/m^2^]	24.5 ± 3.2	23.6 (22.0–26.6)	28.0 ± 4.4	27.2 (25.1–30.3)	< 0.0001
FFM [kg]	42.7 ± 5.3	42.4 (39.4–43.4)	50.5 ± 11.8	47.6 (42.5–60.6)	< 0.0001
FM [kg]	20.1 ± 6.8	18.0 (15.0–24.4)	24.7 ± 7.9	23.0 (21.4–28.3)	0.001
FM%	31.4 ± 6.6	31.6 (28.3–35.8)	32.4 ± 7.2	33.7 (26.7 -37.1)	0.517
WC [cm]	80.2 ± 9.2	77.0 (74.0–86.0)	92.1 ± 11.4	92.0 (85.0–99.0)	< 0.0001
HC [cm]	100.9 ± 6.7	101.0 (95.0–104)	107.4 ± 10.6	107.4 (102.0–110.4)	< 0.001
WHR	0.8 ± 0.1	0.78 (0.74–0.80)	0.9 ± 0.1	0.84 (0.80–0.91)	< 0.0001
LAP	36.7 ± 17.5	29.9 (25.7–43.0)	64.1 ± 28.9	58.4 (49.1–73.8)	< 0.0001
VAI	1.9 ± 0.1	1.6 (1.3–1.9)	1.9 ± 0.6	1.8 (1.5–2.1)	0.202
SBP [mmHg]	128.8 ± 17.5	127 (115.0–128.0)	141.0 ± 16.6	141.0 (129.0–151.0)	0.001
DBP [mmHg]	77.8 ± 7.9	77.0 (71.0–84.0)	83.6 ± 10.0	84.0 (77.5–89.5)	0.003
Heart rate [bpm]	73.4 ± 8.4	72.0 (68.0–76.0)	73.6 ± 9.8	72.0 (66.2–81.7)	0.909

*BMI* body mass index, *FFM* fat-free mass, *FM* fat mass, *WC* waist circumference, *HC* hip circumference, *WHR* waist-hip-ratio, *LAP* lipid accumulation product, *VAI* visceral adiposity index, *SBP* systolic blood pressure, *DBP* diastolic blood pressure, *SD* standard deviation, *Med* median, *IGR* interquartile.

### Hematological variables

The white blood cells count fell within the referential range in all the participants. However, higher values of leukocytes or granulocytes were observed in the group of patients diagnosed with the metabolic syndrome (Table 2). Statistically significant differences were observed between the groups in selected indicators in the red blood cells system, such as RBC (*p* < 0.001), HB (*p* < 0.01) and also in HCT (*p* < 0.001). Platelet values in both groups were within the reference range and no differences were found between the groups.

**Table 2:** Hematological variables.

Variables	Reference values	Without MetS *n* = 37		With MetS *n* = 59		*p*-value
		Mean ± SD	Med (iqr 25%-75%)	Mean ± SD	Med (iqr 25%-75%)	
Leukocytes [10^3^/µL]	5.0–11.6	5.3 ± 1.1	5.2 (4.7–5.9)	5.9 ± 1.5	5.7 (4.9–6.6)	0.086
Lymphocytes [10^3^/µL]	1.3–4.0	1.8 ± 0.5	1.8 (1.4–2.0)	1.9 ± 0.6	1.8 (1.4–2.3)	0.244
Granulocytes [10^3^/µL]	2.4–7.6	3.2 ± 0.9	3.3 (2.6–3.6)	3.7 ± 1.2	3.5 (2.7–4.3)	0.059
LYM%	19.1–48.5	33.6 ± 7.3	34.8 (27.1–39.5)	32.5 ± 6.9	32.1 (27.8–27.7)	0.436
GRA%	43.6–73.4	60.3 ± 7.7	61.0 (54.6–66.3)	61.9 ± 6.7	62.5 (56.2–66.3)	0.292
RBC [10^3^/µL]	F 4.0–5.5	4.3 ± 0.3	4.4 (4.1–4.5)	4.6 ± 0.3	4.6 (4.4–4.8)	< 0.001
	M 4.5–6.6					
HB [g/dL]	F 12.5–16.0	13.5 ± 1.0	13.5 (13.0–13.9)	14.2 ± 1.0	14.2 (13.7–14.6)	0.004
	M 13.5–18.0					
HCT [%]	F 37–47	36.2 ± 2.8	36.1 (35.0–37.7)	38.3 ± 2.7	38.3 (36.6–39.9)	< 0.001
	M 40.0–51.0					
MCV [fL]	F 80–95	83.7 ± 4.9	83.0 (81.0–86.0)	83.3 ± 3.1	83.0 (81.0–86.0)	0.662
	M 80–97					
MCH [pg]	F 27.0–32.0	31.3 ± 2.0	31.1 (30.2–32.0)	30.9 ± 1.3	30.9 (30.0–31.5)	0.132
	M 26.0–32.0					
MCHC [g/dL]	F 32.0–36.0	37.4 ± 0.7	37.5 (36.7–38.0)	37.0 ± 0.7	37.0 (36.60–37.6)	0.014
	M 31.0–36.0					
PLT [10^3^/µL]	150–400	243.9 ± 53.5	240.0 (214.0–288.0)	239.7 ± 52.8	237.0 (217.8–268.0)	0.711

*LYM* lymphocytes, *GRA* granulocytes, *RBC* red blood cells, *HB* hemoglobin, *HCT* hematocrit, *MCV* mean corpuscular volume, *MCH* mean cells hemoglobin, *MCHC* mean corpuscular/hemoglobin concentration, *PLT* platelets, *F* female, *M* male, *SD* standard deviation, *Med* median, *IGR* interquartile.

### Biochemical variables

High levels of TG > 1.7 mmol/L were found in 67.7% of the study individuals whereas high levels of TC > 5 mmol/l and non-HDL > 3.37 mmol/L were recorded respectivelyin 74% and 69.8% of study group. There were no statically significant differences between the groups in total cholesterol, HLD, oxLDL and LDL (Table 3) The only statistically significant differences between the groups were found for triglycerides (*p* < 0.0001). HOMA-IR value > 2.5, indicating insulin resistance, was demonstrated in 52.5% of the MetS group and only in 8% of the group without diagnosed metabolic syndrome. HOMA-IR was significantly correlated with waist circumference (r_s_ = 0.52, *p* < 0.0001), with body weight (r_s_ = 0.50, *p* < 0.0001) and also with FFM (r_s_ = 0.40, *p* < 0.0001).

**Table 3:** Biochemical variables.

Variables	Reference values	Without MetS *n* = 37		With MetS *n* = 59		*p*-value
		Mean ± SD	Med (iqr 25%-75%)	Mean ± SD	Med (iqr 25%-75%)	
TC [mmol/L]	< 5	5.5 ± 1.3	5.6 (4.9–6.4)	5.8 ± 1.2	5.7 (5.0–6.5)	< 0.0001
TG [mmo/L]	< 1.69	1.7 ± 0.3	1.7 (1.6–1.8)	2.0 ± 0.4	1.9 (1.7–2.1)	0.172
LDL [mmol/L]	< 2.6	3.3 ± 0.7	3.3 (2.9–3.8)	3.4 ± 0.8	3.4 (3.0–3.8)	0.509
HDL [mmol/L]	F < 1.3	1.8 ± 0.5	1.8 (1.6–2.0)	1.9 ± 0.3	1.9 (1.6–2.1)	0.259
	M < 1.0					
non-HDL [mmol/L]	< 3.37	3.7 ± 0.9	3.7 (3.2–4.3)	4.0 ± 0.9	3.8 (3.2–4.3)	0.463
oxLDL [ng/L]	–	3513.3 ± 2852.8	1892 (1446–5436)	3483.5 ± 2827	2045 (1361–5114)	0.765
Glucose [mg/dL]	60—115	81.2 ± 8.9	81.9 (75.1–86.8)	103.4 ± 29.7	95.9 (86.1–106.0)	< 0.0001
Insulin [μU/mL]	–	9.9 ± 3.4	9.2 (8.5–9.9)	11.0 ± 3.6	10.0 (8.8–12.8)	0.082
HOMA-IR	< 2.5	2.0 ± 0.8	1.8 (1.7–2.1)	2.9 ± 1.4	2.3 (1.8–3.7)	< 0.001

*TG* triglycerides, *TC* total cholesterol, *LDL* low density lipoprotein, *HDL* high density lipoprotein, *HOMA*-*IR* homeostatic model assessment, *SD* standard deviation, *Med* median, *IGR* interquartile.

### Inflammatory variables

**Figure 1:**
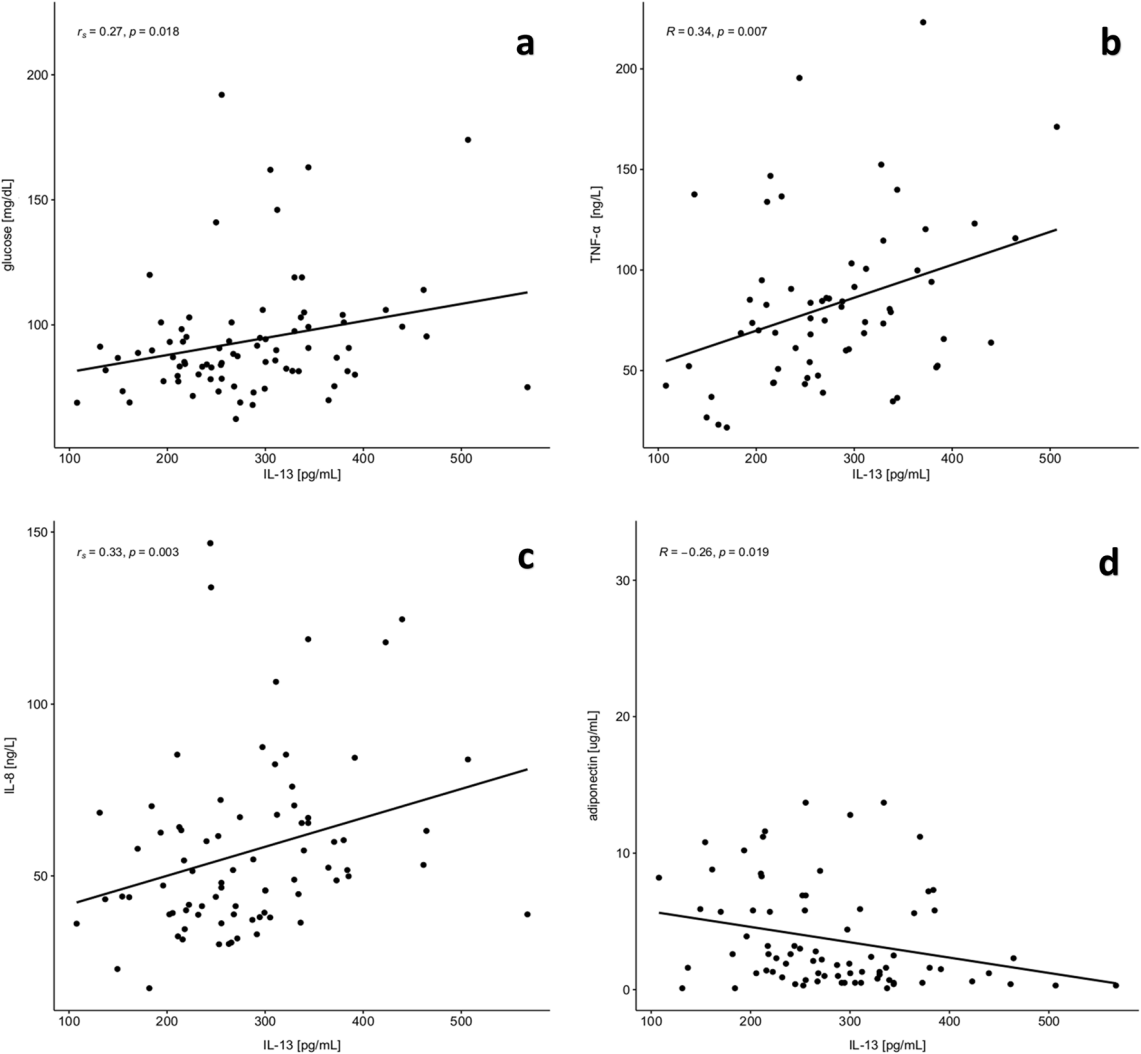
Positive correlation between IL-13 and glucose (**a**), TNF-α (**b**), IL-8 (**c**) and negative correlation between IL-13 and adiponectin (**d**).

**Figure 2:**
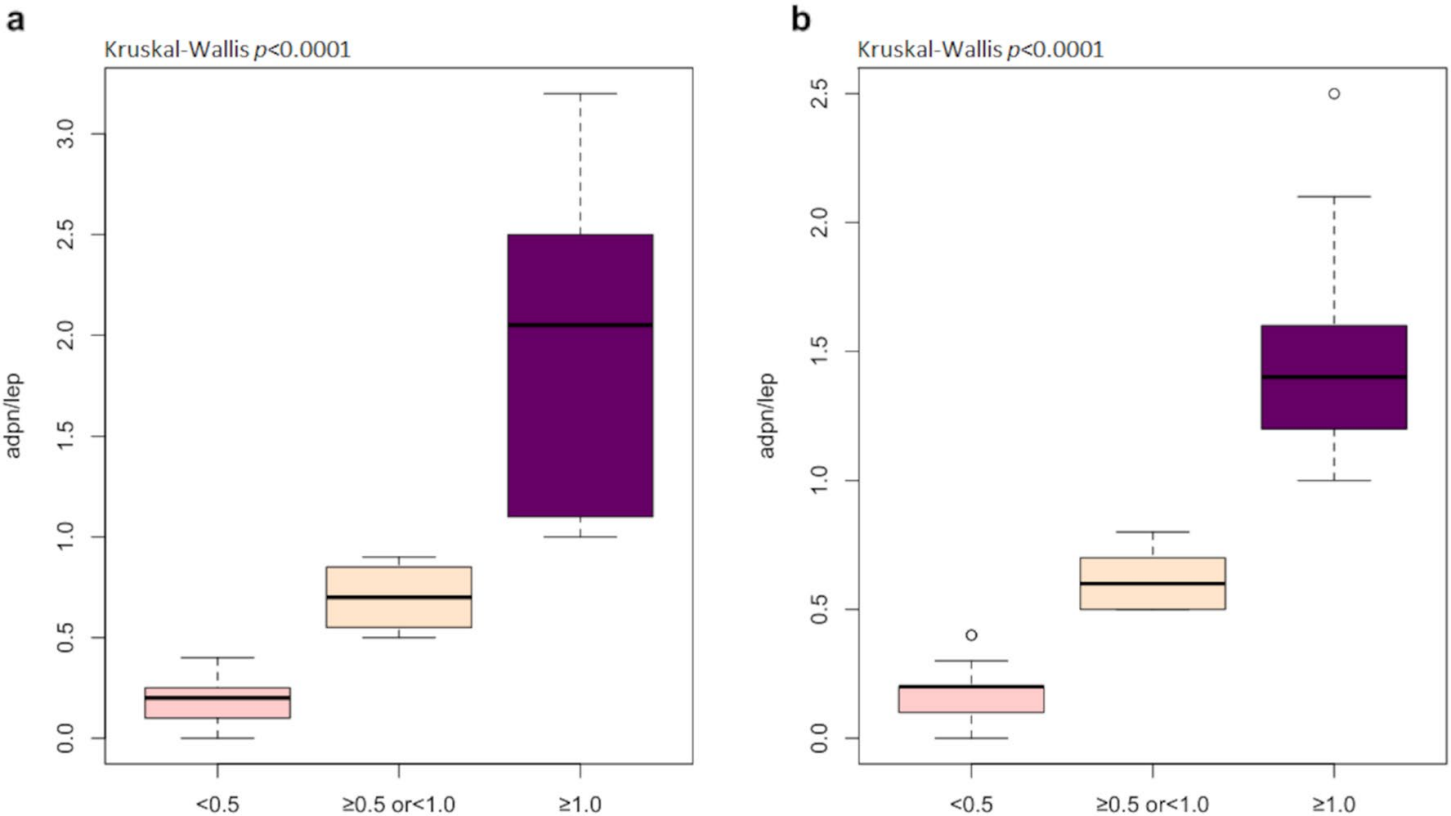
The adiponectin/leptin ratio in group without MetS (**a**) and with MetS (**b**) differentiated to reference ranges for Adpn/Lep (≥ 1.0 normal, ≥ 0.5 or < 1 moderate-medium increased risk, < 0.5 severe increase in cardiometabolic risk).

Adipose tissue has been shown to behave as a highly active endocrine organ owing to its ability to secrete a wide variety of biologically active adipokines, such as TNF-α or IL-6. A classical biomarker of ageing, IL-6, also called “a cytokine for gerontologists”^22^ only tended toward higher values in the MetS group but the difference was not statistically significant (Table 4). A high level of TNF-α was noted in MetS group compared to the group without diagnosed MetS (*p* = 0.027). IL-13 may also participate in low-grade systemic inflammation and insulin resistance and, indeed, higher levels of IL-13 were observed in our group with diagnosed metabolic syndrome. IL-13 exhibited a positive correlation with biochemical parameters like glucose (r_s_ = 0.28, *p* = 0.018) and also with the inflammatory parameters, TNF-α (R = 0.34, *p* = 0.007), IL-8 (r_s_ = 0.33 *p* = 0.003), and negative correlation with adiponectin (R = -0.26, *p* = 0.019) (Fig. 1). A statistically significant difference between groups was also demonstrated in the level of HGMB-1 protein, which may implicate its diagnostic utility in patients with obesity and the metabolic syndrome. Ghrelin was also reported to regulate insulin and glucose metabolism^23^ and a statistically significant difference in its level was observed between our study groups (*p* = 0.029). There were no statistically significant differences between the levels of adiponectin or leptin. Frühbeck et al.^20^, suggested that the Adpn/Lep ratio should be considered as a marker of adipose tissue dysfunction with its value ≥ 1.0 regarded as normal, the ratio ≥ 0.5 or < 1.0 as a moderate-medium increased risk, and the ratio < 0.5 indicating a severe increase in cardiometabolic risk. Surprisingly, in our group without MetS, the Adpn/Lep ratio < 0.5 was noted in 63% of the study individuals and normal values ≥ 1.0 were recorded in only 25%. By contrast, in MetS group 50% of the individuals had the Adpn/Lep ratio < 0.5, 30% fell within the normal range and 20% of the patients presented values ≥ 0.5 or < 1.0, which can suggest a moderate-medium increased risk of cardiometabolic disorders (Fig. 2).

**Table 4:** Inflammatory variables.

Variables	Without MetS *n* = 37		With MetS *n* = 59		*p*-value
	Mean ± SD	Med (iqr 25%-75%)	Mean ± SD	Med (iqr 25%-75%)	
IL-6 [pg/mL]	41.0 ± 26.3	34.8 (19.1–53.6)	45.7 ± 41.4	38.6 (18.7–53.9)	0.567
IL-8 [pg/mL]	56.1 ± 25.2	48.7 (38.3–67.1)	57.1 ± 25.3	52.8 (38.9–65.4)	0.769
IL-13 [pg/mL]	275.1 ± 85.2	269.0 (228.3–315.2)	288.6 ± 87.4	269.0 (231.8–310.1)	0.502
TNF-α [ng/L]	72.3 ± 28.5	73.4 (55.3–84.5)	93.2 ± 43.2	86.1 (65.7–114.6)	0.027
HGMB-1 [ng/mL]	36.3 ± 48.2	12.5 (4.6–44.1)	68.5 ± 59.5	36.1 (11.0–128.3)	0.011
Adiponectin [μg/mL]	4.2 ± 6.0	1.6 (0.8–4.2)	3.7 ± 3.8	2.3 (0.7–5.7)	0.798
Leptin [ng/mL]	5.8 ± 3.0	5.1 (3.4–6.9)	6.4 ± 3.5	5.2 (4.3–7.3)	0.333
Adpn/Lep	0.7 ± 0.8	0.3 (0.2–0.9)	0.7 ± 0.6	0.5 (02.-1.0)	0.655
Ghrelin [pg/mL]	2248.5 ± 2330.5	845.3 (290.3–4604.1)	935.4 ± 1252.3	475.3 (192.8–1045.3)	0.029

*IL-6* interleukin 6, *IL-8* interleukin 8, *IL-13* interleukin 13, *TNFα* tumor necrosis factor α, *HGMB*-*1* high mobility group box-1, *Adpn/Lep* adiponectin to leptin ratio, *SD* standard deviation, *Med* median.

### Assessment of specific variables in the diagnosis of the metabolic syndrome

The results of the ROC analysis of indicators which are likely to be important in the diagnosis of the metabolic syndrome i.e. LAP or TNF-α, ranged between 0.8 and 0.7, which can be considered as a potential diagnostic value for clinical prognosis for patients. The optimal threshold values corresponded to, 45.22 for LAP and 93.4 for TNF-α ng/L (Table 5). The highest AUC, indicating both high sensitivity and specificity, was observed for LAP. For four tested variables, VAI, IL-13, adiponectin and leptin AUC was low, which did not render these factors valuable as diagnostic indicators of the metabolic syndrome in our group. The highest specificity was observed for HOMA-IR (91.7%), ghrelin (89.6%) and TNF-α (89.2), which indicates a low level of false positive results during diagnostic procedures. However, sensitivity values for these variables were fairly low (49.1, 42.5 and 48.9, respectively). This means that more than half of the patients with metabolic syndrome may show lower levels of the markers than the estimated threshold. Consequently, a combined, simultaneous analysis of several factors seems to be the best solution.

**Table 5:** The statistical characteristics of the ROC curve for the univariate logistic model for specific variables (*n* = 96).

Variables	AUC	Cut-off value	Sensitivity (%)	Specificity (%)
VAI	0.495	1.58	49.1	48.6
LAP	0.820	45.22	84.2	78.4
HOMA-IR	0.717	2.48	49.1	91.7
TNF-α [ng/L]	0.765	93.4	48.9	89.2
IL-6 [pg/mL]	0.504	55.3	32.0	78.5
IL-13 [pg/mL]	0.539	336.2	31.9	84.4
HGBM-1 [ng/mL]	0.669	88.2	45.4	85.3
Adiponectin [μg/mL]	0.615	2.06	55.1	59.4
Leptin [ng/mL]	0.550	3.94	84.4	37.5
Ghrelin [pg/mL]	0.649	1992.75	42.5	89.6

*VAI* visceral adiposity index, *LAP* lipid accumulation product, *HOMA*-*IR* homeostatic model assessment, *TNF-α* tumor necrosis factor α, *IL-6* interleukin 6, *HGMB-1* high mobility group box-1, *AUC* the area under the curve, cut-off value the optimal threshold value for clinical stratification.

## Discussion

The aging process is associated with immune changes such as a decline in naïve T lymphocytes or a disproportion in the CD4/CD8 ratio and is referred to as immunosenescence^15,18^. An elderly immune system becomes increasingly more predisposed to chronic inflammatory reactions and less capable of responding to acute and massive challenges posed by new antigens. Overweight or obesity are reported to be on the increase in older adult populations. According to Guarner & Rubio-Ruiz^24^, the increase in the mass of adipose tissue induced a state of systemic inflammation due to an increase in secretory factors (adipokines) derived from pre-adipocytes and from macrophages constituting this tissue. The inflammation significantly contributes to endothelial dysfunction which occurs in cardiovascular diseases (CVDs) developed as a consequence of MetS and diabetes^25^.

Increased adipose tissue, atherogenic dyslipidemia (low HDL and elevated triglycerides), and an increase in blood sugar or high blood pressure ≥ 130/85 are some of the main criteria of the metabolic syndrome that have been introduced by various scientific societies such as the International Diabetes Federation, World Health Organization, the National Cholesterol Education Program (NCEP) Adult Treatment Panel III (ATP III). So far, extensive research has been conducted to determine the factors that influence the metabolic syndrome where attempts have been made to identify the predictors of the metabolic syndrome. Metabolic syndrome predictors can be classified into three categories: anthropometric variables, biochemical variables, and lifestyle^3^. According to the IDF criteria, BMI is one of the basic criteria used to classify the metabolic syndrome. In our study group, MetS patients showed significantly higher anthropometric parameters such as BMI, FFM and FM compared to the patients without MetS most of whom had a normal body weight (68%). Research by Riberto da Costa et al.^26^conducted in the population of Brazil showed that FFM estimated on the basis ofa mathematical model that took into account: age, height, sex, waist and hip circumference was a much better indicator to assess the body composition of Brazilian adults than BAI (ang. *body adiposity index*) which relies only on two variables, i.e. height and hip circumference. The research seems promising, but the validity of the proposed model must be tested in other countries and populationsin order to verify its applicability. As was the case reported by Pouragha et al.^3^, we noted significantly higher WHR values in the group with MetS compared to the group without MetS (*p* < 0.0001), which indicates the diagnostic utility of the above-mentioned indicators. As far as biochemical indices are concerned, statistically significant differences between the groups were observed not only in the glucose level but also in the HOMA-IR index (*p* < 0.001). According to Hrebícek et al.^27^ and Singh & Saxena^28^, the use of two simple homeostatic indicators, HOMA-IR index and quantitative insulin sensitivity check index (QUICKI), can have a comparable diagnostic value for insulin resistance determination as the use of euglycemic hyperinsulinemia. The cut-off value for the HOMA-IR index in our study, regardless of the gender, amounted to 2.48. In the study by Motamed et al.^29^, cut-offs were reported at 2.0 for men and 2.5 for women. Taking into account the predominance of women (78%), the results of our research are also consistent with the results obtained by Fahed et al.^30^, where the AUC and cut-off values were recorded at 2.0 (AUC 0.700) and 2.5 (AUC 0.677) for males and females, respectively. Recently, some attention has been paid to a potential application of new indicators/predictors of the metabolic syndrome assessment, such as LAP which is based on a combination of waist circumference and plasma triglyceride levels^31^. Although the waist-to-height ratio requires only a single anthropometric measurement of waist and height, the use of LAP as a predictor of MetS is more advantageous. Waist circumference measurement is unable to distinguish between visceral and subcutaneous adipose tissue^31^. Visceral adiposity is more strongly associated with a cardiometabolic risks compared with subcutaneous adipose tissue. LAP index has been shown to be a better predictor of diabetes^32^ than body mass index^4^ in cardiovascular risk assessment. Research by Ray et al.^33^ also showed that the new diagnostic indicator of LAP was highly predictive of type 2 diabetes and metabolic syndrome compared to previously described classic anthropometric indicators^34^. In our study, we recorded higher values of the LAP index in patients with MetS (64.1 ± 28.9) compared to the patients without a diagnosed metabolic syndrome (36.7 ± 17.5). The AUC value for LAP was 0.820, which may indicate a significant prognostic value of this index for the assessment of the metabolic syndrome in the studied population of Polish older adults . Our AUC results were similar to the AUC values (0.901) reported in a study of 531 Taiwanese individuals, 105 of whom were diagnosed with MetS according to MS-TW: metabolic syndrome criteria for Taiwanese^31^. Studies conducted on *n* = 552 men from Argentina with an average age of 36.9 ± 10.8 years reported AUC value for LAP at 0.91, while due to considerable age differences between the participants, a significantly higher cut-off value for the LAP index (53.63) was noted when compared to our results (45.22)^35^.

Apart from its thermoregulatory and lipid storing functions, adipose tissue and its endocrine function also provides important information on the development and pathogenesis of the metabolic syndrome. The variety of released adipokines include hormones (e.g. leptin, adiponectin), peptides (e.g. angiotensinogen, apelin, resistin, and plasminogen activator inhibitor 1 (PAI-1), and inflammatory cytokines (IL-6, TNF-α, visfatin, omentin, and chemerin), all of which play a major role in the pathophysiology of insulin resistance and MetS. Insulin resistance and obesity-induced systemic oxidant stress activate downstream inflammatory cascades, leading to tissue fibrosis, atherogenesis, and subsequently CVDs^30^. Studies have shown that the metabolic syndrome is associated with a decrease in ghrelin and adiponectin levels and an increase in leptin levels. The association between low ghrelin and metabolic syndrome is likely to be primarily explained by the relationship with obesity as obese patients with the metabolic syndrome have lower ghrelin levels than nonobese counterparts^10^. In our research a statistically lower value of ghrelin was observed in the MetS group when compared to the group without MetS. The diagnostic usefulness of ghrelin in the assessment of the metabolic syndrome can be proven by the value of AUC 0.649. The obtained test results are similar to those achieved by Sitar-Tǎut et al.^19^, where the AUC for ghrelin was registered at 0.536 for females and 0.718 for males. Research by Yoshinaga et al.^36^ showed that leptin might be the most sensitive marker to predict the metabolic syndrome and a cardiovascular risk in school-age children. In our study, we did not find statistically significant differences between leptin levels, and the AUC value indicated low usefulness of leptin as a predictor in the assessment of the metabolic syndrome. The differences in the obtained outcomes may result from substantial age differences of the respondents as the average age in our study group was 69.2 ± 4.9. In turn, the studies by Falahi et al.^37^ reported that the Adpn/Lep ratio was a better predictor in the assessment of the metabolic syndrome than either of these indicators taken separately. A study of Japanese patients found that Adpn/Lep ratio was significantly and positively associated with the number of components of the metabolic syndrome present, and the ratio was independently associated with each MetS component. In our study, no differences were observed between the Adpn/Lep ratio in the group with MetS and without MetS. Surprisingly, the ratio < 0.5 which indicates a severe increase in cardiometabolic risk^20^ was noted in 50% of our MetS patients and in 63% of the patients without MetS. The observed difference may result from the disproportion between the genders, as in our study women were in the majority in both groups. Some researchers postulate that the difference in the relationship between males and females may result from differences in glucose and lipid metabolism^10^.

The presence of increased adipose tissue has an adverse effect on immune cells. In obese individuals macrophages in the adipose tissue can switch from an anti-inflammatory “M2” (alternatively activated) to a proinflammatory “M1” (classically activated) state and secrete several cytokines (including TNF-α, IL-6, HMGB1)^38^. TNF-α overexpression plays a crucial role in the development of insulin resistance which is an important component of MetS, and some research suggested that TNF-α could be an early predictor for MetS detection^8^. In our study we observed statistically significant differences in TNF-α level (*p* = 0.027) between the study groups. For the first time in Polish population of older adults with the metabolic syndrome, we determined the AUC value as well as the cut-off value for TNF-α. It is noteworthy that in our researchTNF-α showed a good discriminatory capacity for the metabolic syndrome (AUC = 0.765) making this factor a potentially good diagnostic variable. The HGMB-1 protein is not only associated with inflammation but also with insulin resistance and hyperglycemia, and thus also with MetS^39^. Increased levels of HGMB-1 were reported in both mice and diabetic patients^39^. In a cross-sectional study of Chinese adults, the levels of circulating HGMB-1 protein were higher in patients diagnosed with type 2 diabetes (*n* = 76) compared to healthy subjects (*n* = 79). Moreover, it was shown that the level of HGMB-1 was positively correlated with WHR and IL-6^40,41^. In our study, we observed no relationship between the metabolic syndrome and IL-6 and between protein HGMB-1 and IL-6. Nevertheless, the level of HGMB-1 protein was statistically significantly higher in the MetS group (68.5 ± 59.5 ng/mL) when compared to the group without MetS (36.3 ± 48.2 ng/mL). The results of our observations are consistent with those obtained by Wang et al.^40^ who also reported a significant increase of circulating HMGB1 not only in obese individuals but also in patients with type 2 diabetes. Moreover, a relatively high AUC value for the HGMB-1 protein (0.669), higher than for IL-6 (0.504), implicates its diagnostic utility as a predictor of MetS. The research conducted by Arrigo et al.^42^ proved that the HGBM-1 protein may be a new predictor of the metabolic syndrome in obese children (AUC value was equal to 0.992), however, due to a scarcity of scientific reports available, the research must be continued and include larger study groups. Apart from its role in parasite infections, IL-13, a cytokine secreted mainly by activated Th2 lymphocytes, may also play a significant role in the development of metabolic changes, however, clinical findings are still quite controversial^43^. Research by Madhumith et al.^44^ showed that the level of IL-13 decreased in patients with type 2 diabetes compared to the healthy control group. The study by Nestvold et al.^45^, in turn, reported higher values of IL-13 recorded in obese individuals with insulin resistance than in healthy people with a normal body weight. In our study, higher IL-13 values were found in MetS patients in comparison to the healthy group, and the observations were similar to the outcomes reported by Martínez-Reyes et al.^43^, where higher IL-13 values were observed in the insulin resistance group. IL-13 was also studied in reference to low-grade systemic inflammation. In our study, a positive correlation was observed between IL-13 and TNF-α levels (R = 0.34, *p* = 0.007) and between IL-13 and pro inflammatory cytokine IL-8 (r_s_ = 0.33, *p* = 0.003) which may be associated with the role this cytokine plays in the development of low-grade systemic inflammation in individuals with the metabolic syndrome. Studies conducted by Martínez-Reyes et al.^43^ also identified a positive correlation between IL-13 and TNF-α (r = 0.29, *p* = 0.066), but a negative one between IL-13 and the anti-inflammatory cytokine IL-10 (r = − 0.39, *p* = 0.047), so the role of IL-13 in the development of inflammation in people with obesity and insulin resistance still requires further research. It is also worth mentioning that the results of the observed and described correlations only indicate some association, not causation and should be interpreted with caution.

Long-term inflammation is considered to be a significant risk factor for many diseases, including CVDs, the metabolic syndrome and diabetes. The diagnosis of the metabolic syndrome is based on the diagnosis of its constituent disorders. As a risk factor, obesity predisposes to a pro-inflammatory state through an increase in inflammatory mediators such as IL-6, TNF-α or HGBM-1. The search for new predictors of the metabolic syndrome will allow us to diagnose the condition more swiftly and to treat it more effectively.

## Conclusion

For the early prediction and prevention of MetS it is crucial to identify an appropriate index and the corresponding optimal cut-off levels. Studies conducted on Polish older adults have shown high clinical usefulness of the anthropometric indicator (LAP)as well as immunological indicators (TNF-α and HGBMI-1) in predictions of the metabolic syndrome. Moreover, increased body fat tissue in MetS patients is associated with higher levels of inflammatory mediators. The high levels of pro-inflammatory cytokines in patients with higher BMI may be the reason why older adults with MetS are statistically more likely to be obese than controls (people without MetS).

## Data Availability

The datasets used and/or analyzed during the current study are available from the corresponding author on reasonable request.
